# A Reliable Estimate of Visceral Fat Area From Simple Anthropometric Measurements in Chinese Overweight and Obese Individuals

**DOI:** 10.3389/fendo.2022.916124

**Published:** 2022-06-23

**Authors:** Hanying Liu, Di Yang, Shaobo Li, Yunfeng Xiao, Yinfang Tu, Danfeng Peng, Yuqian Bao, Junfeng Han, Haoyong Yu

**Affiliations:** ^1^ Department of Endocrinology and Metabolism, Shanghai Key Laboratory of Diabetes Mellitus, Shanghai Clinical Center for Diabetes, Shanghai Jiao Tong University Affiliated Sixth People’s Hospital, Shanghai, China; ^2^ Department of Radiology, Shanghai Jiao Tong University Affiliated Sixth People’s Hospital, Shanghai, China

**Keywords:** visceral obesity, visceral fat area, visceral adipose tissue, prediction equation, waist circumference, neck circumference

## Abstract

**Objective:**

Visceral obesity, reflected by the amount of visceral adipose tissue (VAT), is associated with multiple chronic diseases and metabolic disorders. The visceral fat area (VFA), measured by MRI, is the ‘gold standard’ for diagnosis of visceral obesity. In this study, a simple model to predict VFA was constructed to facilitate the identification and monitoring of patients who are at high risk of visceral obesity.

**Methods:**

The 721 overweight and obese participants were divided into two groups according to sex, then randomly assigned to derivation and validation cohorts in a 1:2 ratio. Data from the derivation group were used to construct a multiple linear regression model; data from the validation group were used to verify the validity of the model.

**Results:**

The following prediction equations, applicable to both sexes, were developed based on age, waist circumference (WC) and neck circumference (NC) that exhibited strong correlations with the VFA: VFA=3.7×age+2.4×WC+5.5×NC-443.6 (R^2^ = 0.511, adjusted R^2^ = 0.481, for men) and VFA=2.8×age+1.7×WC+6.5×NC-367.3 (R^2^ = 0.442, adjusted R^2^ = 0.433, for women). The data demonstrated good fit for both sexes. A comparison of the predicted and actual VFA in the verification group confirmed the accuracy of the equations: for men, R^2^ = 0.489, adjusted R^2^ = 0.484 and intra-class correlation coefficient (ICC) = 0.653 (p < 0.001) and for women: R^2^ = 0.538, adjusted R^2^= 0.536 and ICC = 0.672 (p < 0.001). The actual and predicted VFAs also showed good agreement in a Bland-Altman plot, indicating the significant correlations of both equations with the actual VFA.

**Conclusions:**

Based on readily available anthropometric data, VFA prediction equations consisting of age, WC and NC were developed. The equations are robust, with good predictive power in both sexes; they provide ideal tools for the early detection of visceral obesity in Chinese overweight and obese individuals.

## Introduction

Obesity is a major public health disease globally, with a prevalence that is steadily increasing in both developed and developing countries. Obesity, especially visceral obesity, is associated with multiple chronic diseases, such as cardiovascular disease (CVD), insulin resistance, type 2 diabetes, and metabolic syndrome (MetS) (1–5). Compared with subcutaneous adipose tissue (SAT), visceral adipose tissue (VAT) expresses larger numbers of genes related to inflammation, oxidative stress, and cytokine production. Increased VAT accumulation is therefore associated with a more severe metabolic, dyslipidaemic, and atherogenic obesity phenotype (2, 3, 6). Accordingly, a fast and simple method for quantifying the regional distribution and content of abdominal fat, especially VAT, can aid in the diagnosis and treatment of obesity.

Numerous techniques for abdominal fat assessment are available for clinical use; these techniques include anthropometry, bioelectrical impedance analysis (BIA), and dual-energy X-ray absorptiometry (DXA) (3, 6). Modern imaging technologies allow accurate and efficient measurement of visceral obesity. Computed tomography (CT) and magnetic resonance imaging (MRI) are currently the ‘gold standard’ methods for direct quantification of the cross-sectional area (CSA) of abdominal fat (e.g., subcutaneous fat area [SFA] and visceral fat area [VFA]) used to classify the degree of abdominal obesity (2, 6, 7).

Because it does not involve ionising radiation, MRI has emerged as a powerful tool for repeatedly quantifying VFA in a non-invasive manner in population-wide studies (7). However, MRI measurements are time-consuming; moreover, imaging is expensive and may not be feasible for extremely obese patients because of scanner-specific weight and space restrictions. As an alternative to MRI, we constructed a simple model to derive predictive equations based on simple clinical variables; our model could be used as an auxiliary method of VFA measurement.

## Subjects and Methods

### Subjects

Our study totally recruited 721 overweight and obese subjects based on body mass index (BMI) from April 2020 to February 2022 at Shanghai Jiao Tong University Affiliated Sixth People’s Hospital, China. Overweight (BMI ≥24.0 to BMI <28.0 kg/m2) and obesity (BMI ≥28.0 kg/m2) were determined in accordance with the standard definitions proposed by the Working Group on Obesity in China. Included subjects were considered generally healthy, as there were no specific patient groups recruited. Pregnant women and those who have recently undergone abdominal surgery were excluded as these may affect the measurement of abdominal fat and/or the anthropometric assessments. All participants were assigned to two groups by gender, including 160 males and 561 females. The two groups were subdivided into derivation and validation cohorts randomly at a ratio of 1:2 for the construction and verification of the model. The study protocol was approved by the Ethics Committee of Shanghai Jiao Tong University and conformed to the Helsinki Declaration. All subjects provided informed consent and underwent abdominal MRI examination, anthropometric and laboratory measurements.

### Anthropometric and Laboratory Assessments

The body weight and height of participants wearing light loose clothes were measured by a digital scale to subsequently calculate BMI = weight (kg)/height squared (m^2^). Circumference measures were conducted by a trained examiner. The tape was placed horizontally and snug to the skin without compressing the soft tissue. Waist circumference (WC) was measured on the midline between the lowest rib margin and the iliac crest. Abdominal obesity was defined as a WC≥90.0 cm for men or a WC ≥85.0cm for women (8, 9). Hip circumference (HC) was measured at the point yielding the maximum circumference over the buttocks. Neck circumference (NC) was measured with head erect and eyes facing forward, horizontally at the upper margin of the laryngeal prominence.

All subjects had a low-fat diet one day before and venous blood samples were taken in the early morning after 8 hours fasting. Laboratory measurements included: alanine aminotransferase (ALT), aspartate aminotransferase (AST), γ-glutamyl transpeptidase (γ-GT), alkaline phosphatase (ALP), prealbumin (PAB), total bile acid (TBA), total bilirubin (TBiL), direct bilirubin (DBiL), blood urea nitrogen (BUN), serum creatinine (Scr), serum uric acid (SUA), retinol-binding protein (RBP), and cystatin C (Cys-C), total cholesterol (TC), triglyceride (TG), high-density lipoprotein cholesterol (HDL-c), low-density lipoprotein cholesterol (LDL-c), serum fasting blood glucose (FBG), hemoglobin A1c (HbA1c), insulin, C-peptide (CP). Hematological and common biochemical examinations were performed according to the manufacturer’s protocol in the same lab using standard laboratory methods.

### Measurement of Body Composition

Abdominal MRI examination was performed using a Philips Achieva 3.0-T magnetic resonance imaging system (Philips Medical Systems, Eindhoven, The Netherlands). Breath-hold fast imaging with a 40-ms repetition time, 2-ms echo time, 50-cm field of view, and 256 × 256 matrix was used to acquire the cross-sectional MR images. One 10-mm slice positioned at the L4 level with a clear outline was selected for analysis using SliceOmatic 5.0 software (TomoVision, Magog, Canada) by a medically trained technician. The psoas CSA, SFA, and VFA were measured using the following steps: regional threshold procedures were first applied using the “Region Growing” mode, after which manual delineation was used to draw borders among different tissues in the “edit mode” when necessary (10). The software calculated different colored areas and expressed the measurements in cm^2^. VFA≥80cm^2^ was defined as visceral obesity.

### Statistical Analysis

All analyses were performed using SPSS version 26.0 (SPSS, Chicago, IL, USA), and a P-value < 0.05 (two-tailed tests) was considered statistically significant. All data were tested for normality using the Kolmogorov–Smirnov test. Continuous variables with normal and non-normal distributions were respectively expressed as mean ± standard deviation (SD) and median (interquartile range, IQR), whereas categorical variables were expressed as percentages. Continuous variables were compared using the Student’s t-test or the Mann-Whitney U-test and categorical variables were compared using the Chi-squared or Fisher’s exact test. The Pearson or Spearman correlation was used to evaluate the relationship between different variables with VAT. Variables correlated with the VFA by correlation analysis were introduced into the stepwise multiple linear regression model within each sex. Thus, the independent predictors of VFA values were identified and screened out to develop the prediction equations. Further, the accuracy of the equations was verified on validation set by reliability analysis and Bland–Altman plot.

## Results

### Baseline Characteristics

A total of 721 subjects meeting the inclusion criteria were recruited, ranging in age from 16 years to a maximum of 65 years. The average VFA value is higher in men than in women (p<0.05). 160 males and 561 females were respectively subdivided into derivation and verification cohorts randomly at a ratio of 1:2. For the male group, there were 53 subjects in the derivation cohort and 107 subjects in the validation cohort; for the female group, there were 187 subjects in the derivation cohort and 374 subjects in the validation cohort. **Table 1** lists the basic characteristics of each cohort. No statistically significant difference was observed between them (p>0.05).

**Table 1:** Baseline Characteristics in the derivation and validation cohorts.

Characteristics	Male group (n = 160)			Female group (n = 561)		
	Derivation cohort (n = 53)	Validation cohort (n = 107)	P-value	Derivation cohort (n = 187)	Validation cohort (n = 374)	P-value
Age (years)	32.0 (27.0, 38.0)	32.0 (26.0, 38.0)	0.79	31.0 (27.0, 37.0)	31.0 (26.0, 35.3)	0.58
BMI (kg/m^2^)	39.7 ± 6.0	40.0 ± 7.0	0.80	36.3 (32.8, 40.7)	36.1 (32.3, 41.1)	0.53
WC (cm)	121.0 (113.5, 134.2)	123.0 (113.0, 135.0)	0.96	110.0 (102.0, 122.0)	110.0 (100.0, 122.0)	0.67
HC (cm)	117.0 (110.0, 126.0)	118.0 (109.0, 127.0)	0.95	114.0 (106.0, 122.0)	113.0 (106.0, 123.0)	0.93
NC (cm)	45.6 ± 3.1	45.2 ± 4.0	0.43	38.5 (36.5, 41.0)	38.3 (36.5, 40.0)	0.34
SBP (mmHg)	141.2 ± 15.9	144.8 ± 17.4	0.20	129.0 (119.0, 143.0)	130.0 (119.8, 143.0)	0.52
DBP (mmHg)	89.3 ± 12.8	92.9 ± 12.1	0.08	84.5 (78.0, 92.0)	85.0 (79.0, 94.0)	0.64
ALT (U/L)	54.0 (38.0, 113.0)	67.0 (38.0, 100.8)	0.76	34.0 (24.0, 64.0)	36.0 (23.0, 62.0)	0.96
AST (U/L)	31.0 (21.5, 57.0)	34.0 (23.0, 51.0)	0.73	22.0 (18.0, 34.5)	24.0 (18.0, 36.3)	0.84
γ-GT (U/L)	51.0 (39.0, 79.5)	53.0 (37.0, 71.0)	0.52	30.0 (21.0, 52.5)	31.0 (21.0, 48.3)	0.79
ALP (U/L)	80.0 (62.5, 99.0)	77.0 (66.8, 91.8)	0.50	72.0 (62.0, 88.5)	73.0 (61.0, 87.0)	0.88
BUN (mmol/L)	4.9 (4.3, 5.8)	5.1 (4.4, 6.0)	0.75	4.6 (4.1, 5.4)	4.6 (3.9, 5.5)	0.27
Scr (mg/dL)	78.1 (68.1, 86.2)	76.0 (68.9, 84.9)	0.55	57.5 (52.0, 65.3)	58.2 (52.0, 64.9)	0.97
SUA (mg/dL)	489.0 (439.5, 547.0)	465.0 (393.0, 533.0)	0.15	392.0 (340.0, 445.0)	388.5 (331.8, 441.3)	0.62
TC (mmol/l)	5.2 ± 1.1	5.2 ± 0.8	0.98	5.2 (4.6, 5.8)	5.2 (4.5, 6.0)	0.65
TG (mmol/l)	2.4 (1.2, 3.2)	2.1 (1.4, 3.0)	0.32	1.5 (1.0, 2.0)	1.5 (1.1, 2.1)	0.24
HDL-c (mmol/l)	1.0 (0.9, 1.2)	1.1 (0.9, 1.3)	0.18	1.2 (1.0, 1.4)	1.2 (1.0, 1.4)	0.67
LDL-c (mmol/l)	3.1 ± 0.7	3.1 ± 0.6	0.58	3.2 ± 0.8	3.2 ± 0.8	0.79
FBG (mmol/l)	5.7 (4.8, 7.9)	5.9 (5.2, 7.2)	0.43	5.5 (5.0, 6.5)	5.4 (4.9, 6.4)	0.42
HbA1c (%)	6.1 (5.6, 7.7)	6.1 (5.6, 7.1)	0.87	5.6 (5.4, 6.2)	5.7 (5.4, 6.4)	0.42
CP (ng/ml)	4.5 (3.3, 5.7)	4.9 (4.0, 5.9)	0.24	3.9 (3.2, 5.1)	3.9 (3.1, 4.9)	0.57
VFA (cm^2^)	221.2 (175.4, 271.3)	232.0 (187.8, 278.4)	0.44	155.3 (116.6, 203.2)	148.0 (115.0, 191.9)	0.35

Data are expressed as the mean ± SD or the median (IQR).

BMI, body mass index; WC, waist circumference; HC, hip circumference; NC, neck circumference; SBP, systolic blood pressure; DBP, diastolic blood pressure; ALT, alanine aminotransferase; AST, aspartate aminotransferase; γ-GT, γ-glutamyl transpeptidase; ALP, alkaline phosphatase; BUN, blood urea nitrogen; Scr, serum creatinine; SUA, serum uric acid; TC, total cholesterol; TG, triglyceride; HDL-c, high-density lipoprotein cholesterol; LDL-c, low-density lipoprotein cholesterol; FBG, fasting blood glucose; HbA1c, glycated hemoglobin; CP, C-peptide; VFA, visceral fat area.

### Correlation Analysis

Bivariate correlation analysis was performed to identify the variables associated with the VFA values in both groups. The correlation coefficients of the potential predictor variables (used to develop the individual equations) with respect to the VFA are given in **Table 2**. In both sexes, age (r = 0.41; P<0.01 for men and r = 0.28; P<0.01 for women), BMI (r = 0.46; P<0.01 for men and r = 0.53; P<0.01 for women), WC measures (r = 0.38; P<0.01 for men and r = 0.49; P<0.01 for women), NC measures (r = 0.47; P<0.01 for men and r = 0.51; P<0.01 for women), FBG (r = 0.53; P<0.01 for men and r = 0.44; P<0.01 for women), HbA1c (r = 0.44; P<0.01 for men and r = 0.47; P<0.01 for women), and CP (r = 0.30; P<0.05 for men and r = 0.39; P<0.01 for women) showed significant associations with VFA. Then we plotted scatter plots for each of these seven variables and the dependent variable (VFA) separately and found a linear relationship between them. Both the independent and dependent variables were continuous variables. Thus, these seven variables that exhibited strong correlations with the VFA were further introduced into the stepwise multiple linear regression model.

**Table 2:** The correlation coefficient of VFA with demographic and anthropometric variables in derivation cohort.

Variables	Male group (n = 53)	Female group (n = 187)
Age	0.41^**^	0.28^**^
BMI	0.36^**^	0.53^**^
WC	0.38^**^	0.49^**^
HC	/	0.36^**^
NC	0.45^**^	0.51^**^
SBP	/	0.41^**^
DBP	/	0.33^**^
ALT	/	0.27^**^
AST	/	0.26^**^
γ-GT	/	0.44^**^
Scr	/	-0.18^*^
SUA	/	0.21^**^
TC	/	0.21^**^
TG	/	0.32^**^
LDL-c	/	0.19^**^
FBG	0.53^**^	0.44^**^
HbA1c	0.44^**^	0.47^**^
CP	0.30^*^	0.39^**^

Statistical significance *P<0.05; **P<0.01.

BMI, body mass index; WC, waist circumference; HC, hip circumference; NC, neck circumference; SBP, systolic blood pressure; DBP, diastolic blood pressure; ALT, alanine aminotransferase; AST, aspartate aminotransferase; γ-GT, γ-glutamyl transpeptidase; Scr, serum creatinine; SUA, serum uric acid; TC, total cholesterol; TG, triglyceride; LDL-c, low-density lipoprotein cholesterol; FBG, fasting blood glucose; HbA1c, glycated hemoglobin; CP, C-peptide.

### Equation Development

**Figure 1:**
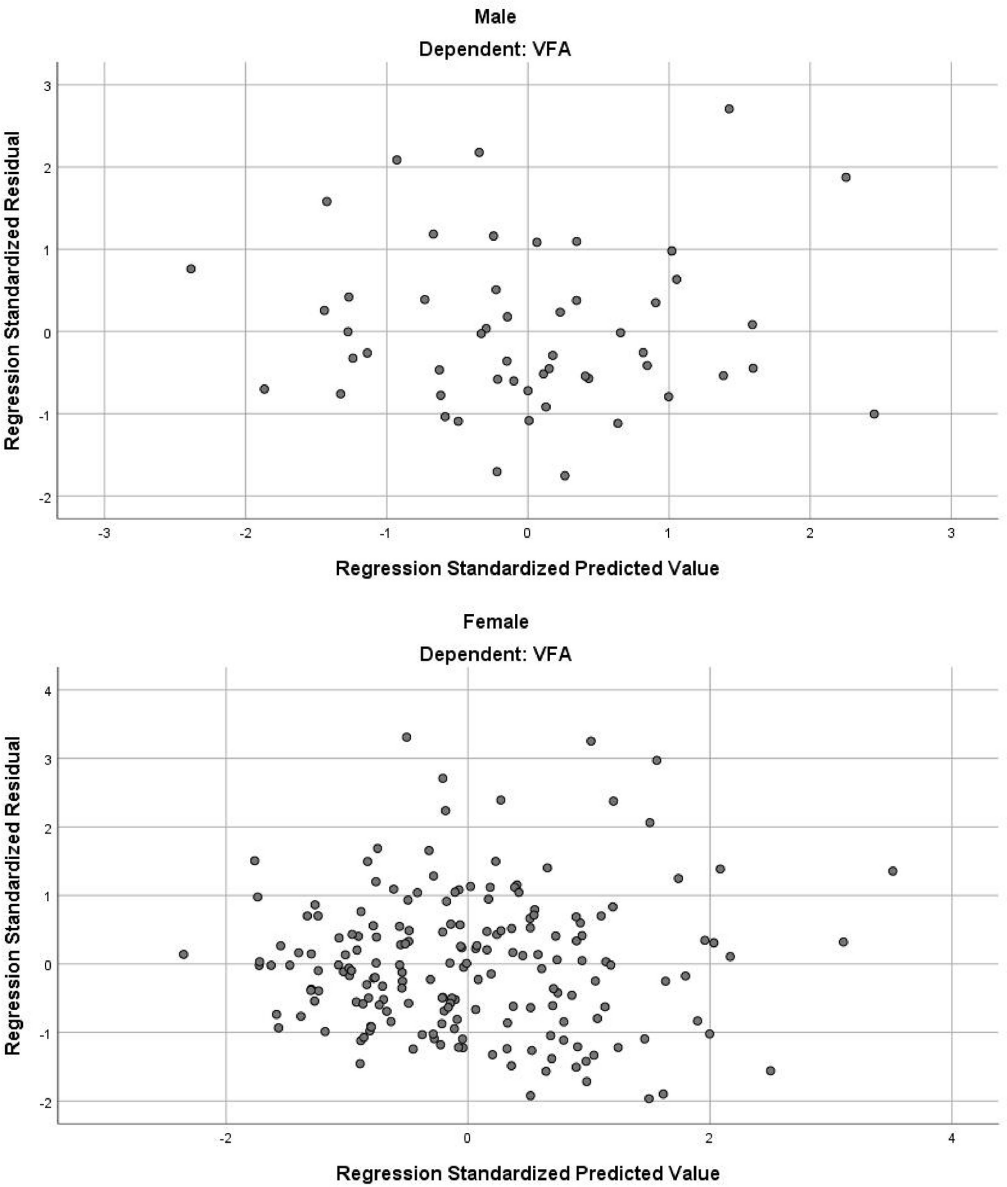
The residual scatter plot of standardized predicted value and standardized residual.

**Figure 2:**
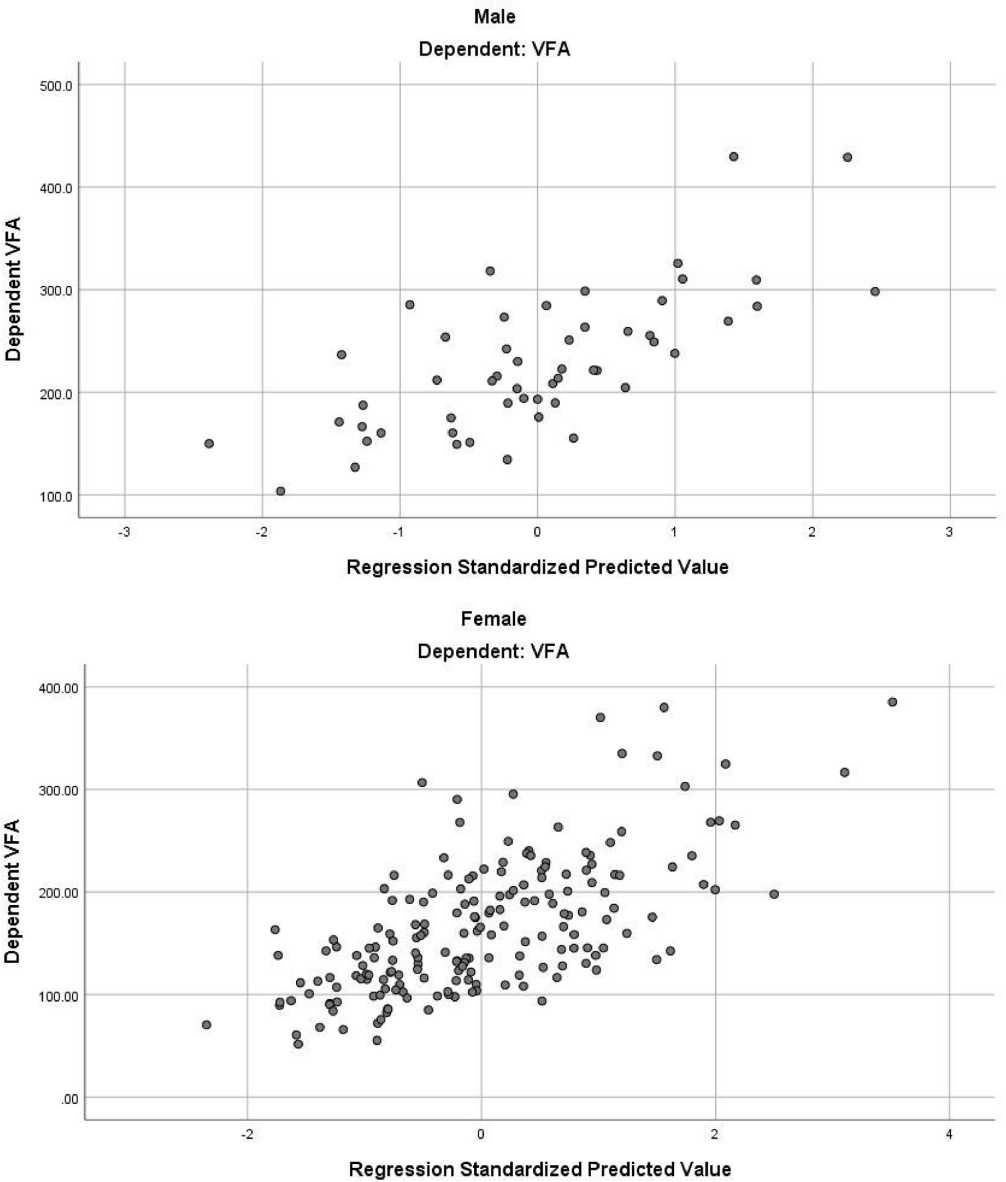
The scatter plot of standardized predicted value and dependent VFA.

In the stepwise multiple linear regression model, the relatively optimal regression equations containing three anthropometric variables (age, WC and NC) for predicting the VFA were derived after multiple variables combination and modification by stepwise regression analysis. These three variables were present in both the male and female groups, but the specific equations were expressed differently. For men, VFA=3.7×Age+2.4×WC+5.5×NC-443.6. The model fitted well with an R^2^ of 0.511 and an adjusted R^2^ of 0.481. **Table 3** provides the regression coefficient and 95% confidence interval (CI) of each variable. The Durbin-Watson test of model residuals was 2.296, indicating that there was no significant correlation between the residuals. Based on the collinearity analysis, the tolerances were more than 0.5 and the variance inflation factor (VIF) values were less than 2, showing that there was no covariance among the independent variables. For women, VFA=2.8×Age+1.7×WC+6.5×NC-367.3 (R^2^ = 0.442, adjusted R^2 ^ = 0.433). As shown in **Table 3**, the model also demonstrated good fit in female group. In addition, **Figure 1** presents the residual scatter plots with the standardized predicted value on the X axis and the standardized residual on the Y-axis, to better appreciate the differences between values predicted and observed (i.e., the residuals) against the values predicted. The scatter points were randomly distributed and the slope was almost zero, which showed the variance homogeneity of the residuals. There was a linear trend in both sexes based on the scatter plots of the standardized predicted value and dependent VFA (**Figure 2**). We also observed that the residuals were approximately normally distributed through the histograms and normal P-P plots of the residuals. All the above results showed that the equations we established satisfied the assumptions of linear regression model and were statistically significant.

**Table 3:** The establishment of new equations in male and female groups respectively.

Gender	Equation	R^2^	Adjusted R^2^	Durbin-Watson test	Variables	Coefficients	95%CI	P-value	Tolerance	VIF
Male	VFA=3.7×Age+2.4×WC+5.5×NC-443.6	0.511	0.481	2.296	Age	3.74	2.17, 5.30	0.000	0.907	1.103
					WC	2.39	1.15, 3.64	0.000	0.677	1.477
					NC	5.53	0.42, 10.64	0.035	0.735	1.361
					Constant	-443.59	-658.37, 228.81	0.000	/	/
Female	VFA=2.8×Age+1.7×WC+6.5×NC-367.3	0.442	0.433	2.176	Age	2.84	1.89, 3.78	0.000	0.982	1.019
					WC	1.69	1.07, 2.30	0.000	0.641	1.561
					NC	6.50	3.69, 9.32	0.000	0.650	1.538
					Constant	-367.28	-462.36, -272.20	0.000	/	/

WC, waist circumference; NC, neck circumference.

### Verification of Equations

**Figure 3:**
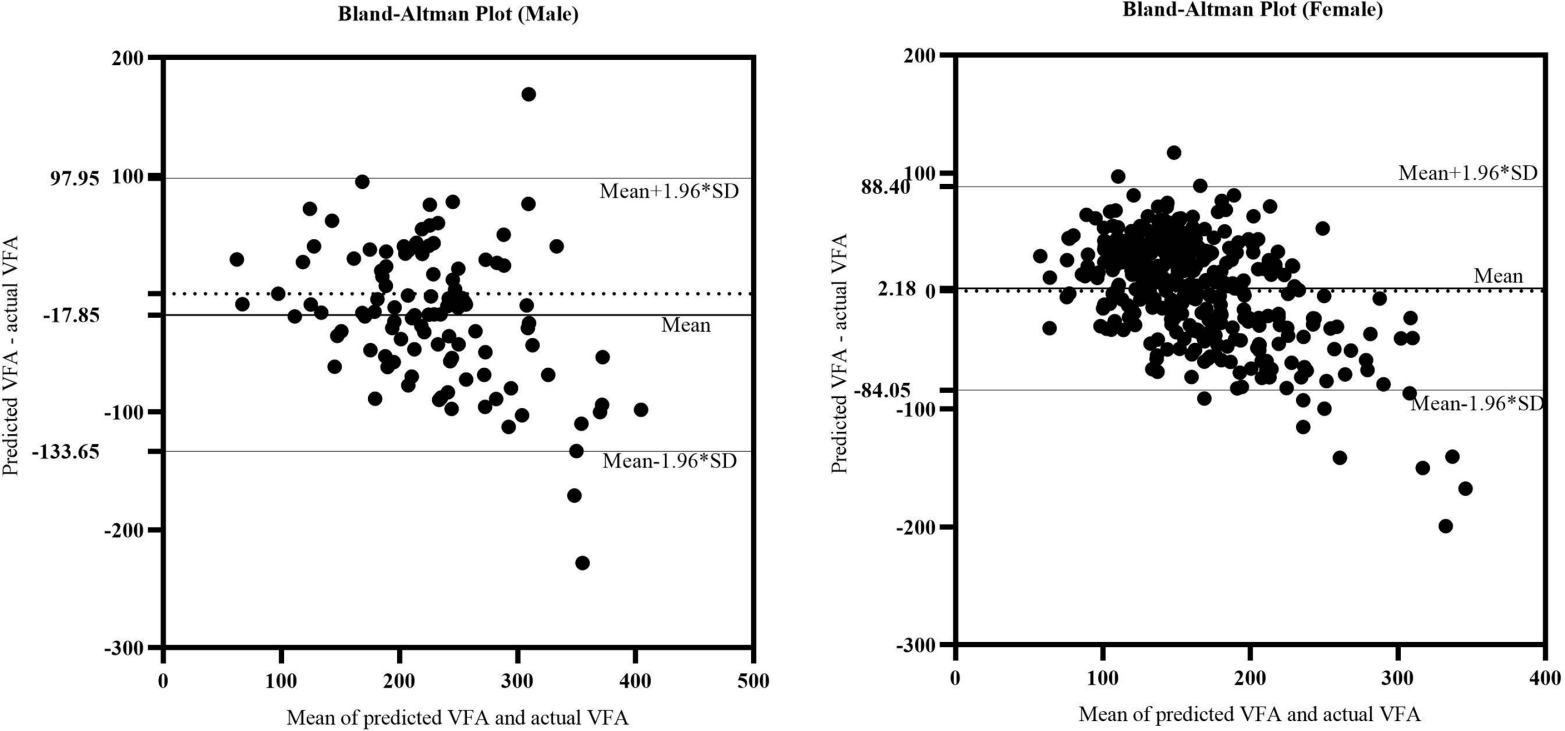
The Bland-Altman plot of actual CAP and predicted CAP. The upper and lower horizontal solid lines in the picture represented the 95% limits of agreement. The middle horizontal solid line in the middle represented the average value of the difference. The horizontal dotted line indicated the position where the average value of the difference was zero.

We further verified the accuracy of the equations in the validation cohorts respectively. A comparison of the predicted and actual VFA in the verification group confirmed the accuracy of the equations: for men, R^2^ was 0.489 and the adjusted R^2^ was 0.484; for women, R^2^ was 0.538 and the adjusted R^2^ was 0.536. On average, predicted and actual VFA values were 224 and 232 cm^2^ in men and 159 and 148 cm^2^ in women. The consistency of predicted and actual VFA on the same subject was evaluated using reliability analysis. In two-way random model and absolute agreement type, the values of intra-class correlation efficient (ICC) (single measures) were 0.653 for men and 0.672 for women (p<0.001). **Figure 3** is a Bland–Altman plot showing that in both sexes, the actual and predicted VFAs also showed good agreement; most of the differences were within the 95% limits of agreement. In addition, the mean value of the differences was close to zero. Therefore, it can be assumed that the predicted VFA showed a significant and high consistency with the actual VFA in both equations.

Previous researches have given different VFA prediction equations for two sexes as well: i) Bonora et al. : VFA=6.37×WC-453.7 (for men) and VFA=2.62×Age+4.04×WC-370.5 (for women) (11); ii) Brundavani et al. : VFA=1.09×weight+6.04×WC-2.29×BMI-382.9 (for men) and VFA=-0.86×weight+5.19×WC-278 (for women) (12); iii) Goel et al. : VFA=0.169×Age+5.7809 × BMI-4.4106×HC+4.342×WC+6.9548 (for men) and VFA=0.169×Age+5.7809 × BMI-4.4106×HC+4.342×WC+16.2966 (for women) (13). We also validated these pre-existing equations separately in our validation cohorts, the corresponding R^2^ and adjusted R^2^ of them were all less than 0.45 (**Table 4**). The ICC values of their equations were less than ours likewise.

**Table 4:** **|**The validation of the equations from our study and other studies.

Equation		R^2^	adjusted R^2^	ICC
Our study				
male	VFA=3.7×Age+2.4×WC+5.5×NC-443.6	0.489	0.484	0.653
female	VFA=2.8×Age+1.7×WC+6.5×NC-367.3	0.538	0.536	0.672
Bonora et al. (11)				
male	VFA=6.37×WC-453.7	0.284	0.277	0.348
female	VFA=2.62×Age+4.04×WC-370.5	0.411	0.409	0.636
Brundavani et al. (12)				
male	VFA=1.09×Weight+6.04×WC-2.29×BMI-382.9	0.269	0.262	0.199
female	VFA=-0.86×Weight+5.19×WC-278	0.273	0.271	0.374
Goel et al. (13)				
male	VFA=0.169×Age+5.7809 × BMI-4.4106×HC+4.342×WC+6.9548	0.313	0.307	0.524
female	VFA=0.169×Age+5.7809 × BMI-4.4106×HC+4.342×WC+16.2966	0.318	0.316	0.403

WC, waist circumference; NC, neck circumference; BMI, body mass index.

Therefore, the above results suggested that both sets of equations obtained by our stepwise regression analysis have excellent predictive performance and high application value in clinical promotion. For medical institutions where MRI examination of body composition is not available, clinicians can estimate VFA values more quickly and accurately by measuring waist and neck circumferences of each patient and substituting these simple anthropological indicators into the equations.

## Discussion

Visceral obesity, characterised by dysfunctional adipose tissue storage and ectopic triglyceride accumulation in several sites including the liver (4), increases the risks of metabolic disorders and CVD (2–4). Quantitative assessment of visceral obesity is therefore essential to determine the potential risks and establish an accurate prognosis. Because VAT is located in the abdominal cavity, under the surrounding abdominal and back muscles, it is difficult to measure. MRI has been used to quantitatively measure abdominal fat CSA and is considered the ‘gold standard’ method for assessment of VAT. However, while MRI is non-invasive, radiation-free, repeatable, and applicable to all age groups, its cost and time-consuming nature limit its wider adoption for large-scale screening or routine clinical practice. In light of these limitations, we developed a simple VFA prediction linear regression model to facilitate the early detection and quick assessment of visceral obesity.

The distributions and functions of adipose tissue vary between men and women because of differential sex hormone effects. On average, VAT mass is higher in men than in women, regardless of age (14). Estrogen promotes the accumulation of SAT in women and the deposition of visceral fat in men (15); in contrast, androgen excess is presumed to favour the expansion of VAT (16). Generally, men tend to accumulate more VAT, resulting in the classic ‘apple’ body shape that is also associated with an increased cardiometabolic risk. In contrast, premenopausal women typically accumulate more SAT on the hips, thighs, and buttocks; they are thus protected against the negative effects associated with obesity and MetS (15, 16). Considering these sexual differences, the derivation of corresponding VFA prediction equations required division of our study participants into two sex-based groups to allow the construction of separate prediction models. Through stepwise multiple linear regression analysis, the relatively optimal equations were then derived by determining three anthropometric variables: age, WC, and NC.

VAT deposition increased with age in both men and women. The increase was particularly large in postmenopausal women, in whom a decline in estrogen levels is associated with the accumulation of visceral fat (15). The hormonal changes are accompanied by an age-related shift in fat distribution (from subcutaneous to visceral) (17), which contributes to the age-related increase in VAT in both sexes (14). A stronger relationship between age and VAT before than after the age of 70 has been reported (18); a progressive increase in the mean VAT with age until approximately 65–70 years, followed by a gradual decrease thereafter (14), has also been reported. In a study population from the United Arab Emirates, Yoo et al. identified cut-off values of CT-measured VAT to predict MetS: 132.0 cm^2^ in both sexes for individuals aged < 50 years, and 173 cm^2^ in women and 124.3 cm^2^ in men for individuals aged > 50 years (19). In the study by Brundavani, age did not contribute to the prediction of VAT, perhaps because the study population was between 40 and 80 years of age; the biological effects of peripheral fat mobilization on centralization and internalization had already occurred and age no longer had a significant effect (12).The predictive equations derived from our study clearly demonstrate that VAT increases with age. However, because our participants were not older than 65 years, an age cut-off for VAT decline could not be determined.

Waist circumference (WC) has been commonly used in the clinical setting as a rough estimate of visceral adiposity (2). Although WC cannot accurately distinguish between visceral and subcutaneous fat deposits (4, 20, 21), it remains an extremely simple and inexpensive method currently that correlates with visceral adiposity (2, 22). Jia et al. performed a receiver operating characteristic (ROC) curves analysis indicating that WC had the best accuracy in predicting visceral obesity in comparison with BMI and waist-to-hip ratio (WHR) (23). However, there is no consensus regarding the optimal anatomical site to measure WC. In several studies, the most practical measurement protocols for clinical use were (2, 20–22, 24): the superior border of the iliac crest, as described in the National Institutes of Health guidelines; below the lowest rib; the midpoint between these two sites, as recommended by the World Health Organization and International Diabetes Federation guidelines; minimal waist; and umbilicus. The higher mean WC value for men indicated a different pattern of body fat distribution than the pattern present in women. Previous studies reported absolute differences in WC measurements obtained at different sites, especially in women. For example, Bosy-Westphal et al. found that WC below the lowest rib was strongly associated with VAT and cardiometabolic risk factors in women (21). In the study by Pinho et al., minimal waist was significantly correlated with VAT (r = 0.70) and with a larger spectrum of cardiometabolic parameters among men (20). According to Seimon et al., WC measurements obtained at the midpoint between the lowest rib and iliac crest and at the minimal site were more closely correlated with MRI-measured VAT than were measurements at the umbilicus (r = 0.581, 0.563, and 0.390, respectively; p < 0.001) (24). The authors thus recommended minimal waist measurement for effective estimation of VAT in postmenopausal obese women; notably, it does not require the palpation and the identification of two bony anatomical landmarks. Likewise, Johnson et al. proposed that WC measured at the narrowest site and at the midpoint between lowest rib and iliac crest were most strongly and consistently associated with the MetS and metabolic risk factors (25). A systematic review also indicated that WC measured at midline between the lowest rib and iliac crest was the most valid and reliable measure to assess visceral fat content and changes in visceral fat over time in both sexes (26). Therefore, the midpoint with a relatively high correlation with VAT was selected as the WC measurement site in our study. The different studies mentioned above suggested that valid comparisons among studies will require standardization of WC measurement protocols and the influence of SAT should also be considered when assessing WC measurements.

Neck circumference (NC) is a novel, easily accessible, and replicable anthropometric measurement that reflects ectopic fat distribution in the neck. A significant correlation between NC and VAT in both men and women has been reported in several studies (27–30). Li et al. found that neck fat area was positively associated with abdominal VAT in both sexes, which may explain the relationship between NC and VAT (28). Based on an analysis of ROC curves, Luo et al. determined that the areas under the curve for the ability of NC to determine visceral adiposity (VFA ≥ 80 cm^2^) were 0.781 for men and 0.777 for women in China. The authors also obtained optimal cut-offs for identifying visceral obesity: ≥ 38.5 cm for men (sensitivity of 56.1% and specificity of 83.5%) and ≥ 34.5 cm for women (sensitivity of 58.1% and specificity of 82.5%) (29). Their findings indicated no differences in the sensitivity and specificity of NC vs. WC for the diagnosis of metabolic disorders. Nonetheless, as an emerging metric, NC has not yet been applied worldwide like WC. NC is a practical clinical predictor of VAT because it uses an explicit landmark, has low variability, and is minimally affected by breathing, diet, and position. Therefore, it could be promoted as a feasible measure of visceral obesity in parallel with WC in large-scale population studies and should be regularly used to monitor individuals with increased visceral adiposity.

We also validated several prediction equations obtained in previous studies and found that the validity of the equations established in our study was higher for a few reasons. First, our sample size was much larger, which improved the accuracy of our equations. Second, the participants in the previous studies came from Italy, Tirupati, and North India; the corresponding equations performed poorly in our Chinese study population. Third, our study specifically focused on overweight and obese individuals, whereas the other studies also included individuals of normal weight.

The key strengths of this study were its large sample size and the identification of NC as an important contributor to VFA. The limitations included the smaller proportion of men than women and the single-center design with only Asian participants. Therefore, the equations require further external validation in different ethnic groups and centers.

## Conclusion

The equations developed in this study to predict VFA consist of simple anthropometric measures (age, WC and NC). Their demonstrated validity supports their use as surrogate tools to discern and monitor high-risk individuals with visceral obesity.

## Data Availability Statement

Due to the privacy of patients, the data related to patients cannot be available for public access but can be obtained from the corresponding author on reasonable request approved by the institutional review board of Shanghai Jiao Tong University Affiliated Sixth People’s Hospital. Requests to access these datasets should be directed to yuhaoyong111@163.com.

## Ethics Statement

The studies involving human participants were reviewed and approved by Ethics Review Committee of Shanghai Jiao Tong University Affiliated Sixth People’s Hospital. The patients/participants provided their written informed consent to participate in this study.

## Author Contributions

HL drafted the manuscript. DY and YT performed the statistical analysis. DY, DP and SL drafted the figure and legend. YX manipulated MRI. YB, JH, and HY designed the outline of the topic and helped on revising the manuscript. All authors contributed to the article and approved the submitted version.

## Funding

This study was supported by grants from the Clinical Research Plan of SHDC (SHDC2020CR1017B), National Key Research and Development Project of China (2016YFA0502003), National Natural Sciences Foundation of China (8217033843), Shanghai Municipal Education Commission-Gaofeng Clinical Medicine Grant Support (20191920), Shanghai Key Clinical Center for Metabolic Disease (2017ZZ01013) and Shanghai Municipal Key Clinical Specialty.

## Conflict of Interest

The authors declare that the research was conducted in the absence of any commercial or financial relationships that could be construed as a potential conflict of interest.

## Publisher’s Note

All claims expressed in this article are solely those of the authors and do not necessarily represent those of their affiliated organizations, or those of the publisher, the editors and the reviewers. Any product that may be evaluated in this article, or claim that may be made by its manufacturer, is not guaranteed or endorsed by the publisher.
